# *Loa loa* Infection in Pregnant Women, Gabon

**DOI:** 10.3201/eid2105.141471

**Published:** 2015-05

**Authors:** Ghyslain Mombo-Ngoma, Jean Rodolphe Mackanga, Arti Basra, Meskure Capan, Rella Zoleko Manego, Ayôla Akim Adegnika, Felix Lötsch, Maria Yazdanbakhsh, Raquel González, Clara Menendez, Barthelemy Mabika, Pierre Blaise Matsiegui, Peter G. Kremsner, Michael Ramharter

**Affiliations:** Universität Tübingen, Tübingen, Germany (G. Mombo-Ngoma, J.R. Mackanga, A. Basra, M. Capan, R.Z. Manego, A.A. Adegnika, P.B. Matsiegui, P.G. Kremsner, M. Ramharter);; Centre de Recherches Médicales de Lambaréné, Lambaréné, Gabon (G. Mombo-Ngoma, J.R. Mackanga, A. Basra, M. Capan, A.A. Adegnika, F. Lötsch, P.G. Kremsne, M. Ramharter);; Ngounié Medical Research Centre, Fougamou, Gabon (G. Mombo-Ngoma, R.Z. Manego, P.B. Matsiegui);; Université des Sciences de la Santé, Libreville, Gabon (G. Mombo-Ngoma, B. Mabika);; Leiden University Medical Centre, Leiden, the Netherlands (A.A. Adegnika, M. Yazdanbakhsh);; Medical University of Vienna, Vienna, Austria (F. Lötsch, M. Ramharter);; Hospital Clinic Universitat de Barcelona, Barcelona, Spain (R. González, C. Menendez)

**Keywords:** Loa loa, filarial, pregnancy, epidemiology, parasites, zoonoses, Africa, Gabon, helminths, eye worms

**To the Editor:** *Loa loa*, the African eye worm, is a filarial pathogen of Central African rainforest regions. As of 2013, it had affected an estimated 2–3 million persons in Central Africa (*1*,*2*). Adult worm migrations in humans may intermittently cause Calabar swelling, and microfilariae are commonly found in blood and body fluids. Loiasis is a chronic infection persisting for many years; a considerable proportion of women in loiasis-endemic regions are infected during gestation. To date, the epidemiology of loiasis in pregnant women has not been investigated, and the effects of loiasis on maternal and fetal health outcomes are unknown. We investigated the epidemiology of loiasis in a cohort of pregnant women participating in a drug trial for preventing malaria during pregnancy.

This study was conducted at the Centre de Recherches Médicales de Lambaréné, Albert Schweitzer Hospital, Lambaréné, Gabon, and at the Ngounié Medical Research Centre, Fougamou, Gabon, during September 2009–April 2012 (*3*). The filarial pathogens *L. loa* and *Mansonella perstans* are endemic to the study region, which is in the equatorial rainforest, and malaria is hyperendemic in the region (*1*,*4*). Study participants were HIV-negative pregnant women in a clinical trial assessing intermittent preventive treatment of malaria during pregnancy (clinical trials identifier: Malaria in Pregnancy Preventive Alternative Drugs [MiPPAD]; NCT00811421) (*5*). Pregnant women were recruited before their third trimester. After providing written informed consent, they were randomly allocated to receive treatment with either sulfadoxine/pyrimethamine or mefloquine. Ethical clearance was obtained from the Comité d’Ethique Regional Indépendent de Lambaréné.

Women for whom >1 microscopic examination of blood revealed microfilariae during the course of their pregnancy were classified as *L. loa* infected. Women were considered afilaremic if >2 blood examination results were negative. Microfilariae were detected by examination of thick and thin blood smears or by saponin leukoconcentration. Examination for placental infection was performed by impression smear of a fresh placental biopsy. Baseline demographic and anthropometric characteristics were recorded at the first antenatal visit and at delivery. Data were double-entered into an electronic database for statistical analysis (STATA/SE 12.1; StataCorp LP, College Station, TX, USA).

Of 1,184 women participating in the antimalarial drug trial, 1,004 contributed data for our analysis. Women who had no record of delivery (n = 120) or who had multiple births (n = 60) were excluded. Of these 1,004 women, *L. loa* microfilariae were found in peripheral blood of 179 (18%); of those, microfilariae were found in placental blood of 24 (13%). No microfilariae were found in the placenta of women with amicrofilaremic peripheral blood. Loiasis prevalence was higher among older women (>30 years of age) than among adolescents (14–17 years of age; odds ratio 2.1, 95% CI 1.2–3.9). Microfilaremia was more common among multigravid women than among primigravid women (p = 0.06) but was not associated with other maternal baseline characteristics or with low infant birthweight, preterm births, or adverse delivery outcomes (Table). No histologic evidence of intervillous inflammation, infarcted areas, or chorioamnionitis was observed in the placenta of women with loiasis, and no microfilariae were observed in any examined cord blood samples.

**Table:** Baseline characteristics of microfilaremic and amicrofilaremic pregnant women, Gabon, September 2009–April 2012

**Characteristic**	**Microfilaremic, no. (%), n = 179**	**Amicrofilaremic, no. (%), n = 825**	p value*
**Age, y, n = 1,004**			0.156
**14–17**	17 (9.5)	129 (15.6)	
18–20	43 (24.0)	191 (23.2)	
21–24	40 (22.3)	168 (20.4)	
25–30	34 (19.0)	175 (21.2)	
31–49	45 (25.1)	162 (19.6)	
**Gravidity, n = 1,004**			0.057
First pregnancy	40 (22.3)	214 (25.9)	
1–3 previous pregnancies	73 (40.8)	381 (46.2)	
**>4 previous pregnancies**	66 (36.9)	230 (27.9)	
**Literacy, n = 1,004**			0.151
Yes	141 (78.8)	687 (83.3)	
No	38 (21.2)	138 (16.7)	
**Delivery outcome, n = 1,004**			0.432
Live birth	172 (96.1)	781 (94.7)	
Stillbirth or abortion	7 (3.9)	44 (5.3)	
**Maternal malarial infection at delivery, n = 867†‡**			0.453
Yes	6 (3.7)	36 (5.1)	
No	156 (96.3)	669 (94.9)	
**Anemia at delivery, hemoglobin <11 g/dL, n = 903†**			0.364
Yes	77 (46.1)	368 (50.0)	
No	90 (53.9)	368 (50.0)	
**Premature delivery, n = 886†**			0.082
Yes	14 (8.5)	36 (72.0)	
No	151 (91.5)	685 (95.0)	
**Low birthweight, n = 905†**			0.465
** Yes**	24 (14.3)	90 (12.2)	
No	144 (85.7)	647 (87.8)	

*By χ^2^ test. †Maternal malarial infection, anemia, prematurity, and birthweight were assessed for live births only in this analysis. ‡Low birthweight occurred in 7 (17%) of 41 babies born to malaria-infected mothers compared with 98 (14%) of 721 babies born to non–malaria-infected mothers.

In this study, we attempted to characterize the epidemiology of *L. loa* infection during pregnancy in a highly *L. loa–*endemic region of Central Africa. Microfilaremia was associated with the women’s age, a finding indicating that prevalence increases because of the long duration of infection and continued exposure. The age-related increase in the prevalence of *L. loa* infection aligns with previously reported prevalence and contrasts with age-related prevalence of other parasitic infections (*6*,*7*). Anecdotal evidence suggests the potential of *L. loa* worms to invade the placenta (*8*). In this systematic investigation, microfilarial invasion of the placenta occurred in 13% of microfilaremic patients. However, histopathologic analysis showed no evidence for pathologic alterations of the placenta, and risk for adverse birth outcomes did not increase. Transgression of microfilariae into cord blood was not observed.

This study has limitations. First, misclassification of occult infection is possible because infection status was classified on the basis of the presence of microfilaremia. Also, antimalarial drugs routinely administered during pregnancy may have influenced the course of loiasis, as has been shown for other helminth infections (*9*,*10*). Further, the observational study design creates difficulties in establishing a causal relationship between infection status and birth outcomes because of possible confounding factors. Additional research is needed to disentangle the association among pregnancy outcomes, socioeconomic conditions, and pathophysiologic consequences of *L. loa* infection in pregnant women.

Results of this prospective study show a high prevalence of loiasis among pregnant women in a loiasis-endemic region in Central Africa. The invasion of microfilariae into the intervillous space of the placenta is a newly described feature of pregnancy-associated loiasis. These data can be used as a starting point for further epidemiologic and clinical research activities investigating this neglected filarial infection in pregnant women in Central Africa.
